# The additional genetic diagnosis of homozygous sickle cell disease in a patient with Waardenburg-Shah syndrome: a case report

**DOI:** 10.1186/s13256-018-1953-z

**Published:** 2019-01-13

**Authors:** Angela E. Rankine-Mullings, Graham Serjeant, Zachary Ramsay, Neil A. Hanchard, Monika Asnani

**Affiliations:** 1 Sickle Cell Trust (Jamaica), 14 Milverton Crescent, Kingston 6, Jamaica; 2 Sickle Cell Unit, Caribbean Institute for Health Research, The University of the West Indies, Kingston 7, Jamaica; 3 Department of Molecular and Human Genetics, Baylor College of Medicine, Houston, TX 77030 USA

**Keywords:** Sickle cell disease, Waardenburg-Shah syndrome, Constipation, Deafness, Blue eyes

## Abstract

**Background:**

It is important that multiple genetic diagnoses are not missed. This case report describes the clinical features and management of a patient with co-inheritance of Waardenburg syndrome type 4 or Waardenburg-Shah syndrome, an extremely rare disease, and homozygous sickle cell disease not uncommon in the Caribbean. This case is unusual as it may be the first documented case of the co-inheritance of both these diseases. Given the commonality of sickle cell and related hemoglobinopathies, such combined disorders are likely to be under-reported. Importantly, reporting this case will add to the medical literature as it will raise awareness of the phenotypic manifestations of this disorder.

**Case presentation:**

A 54-year-old Afro-Caribbean woman had a delayed diagnosis of homozygous sickle cell disease at 7 years of age by hemoglobin electrophoresis. The complications of sickle cell disease she experienced included bone pain, a chronic right leg ulcer, avascular necrosis of her left hip, and symptomatic cholelithiasis. This diagnosis was preceded by an earlier diagnosis of Waardenburg syndrome. The basis for the diagnosis of Waardenburg-Shah syndrome was the presence of pigmentary disturbances of her eyes (hypoplastic blue irides), congenital sensorineural hearing loss, and Hirschsprung’s disease. She was mute and complained of chronic constipation which required disimpaction on several occasions. She attended a school for the deaf and communicated via writing. A Duhamel procedure bypassing her rectum was performed at age 9. She died following an admission for acute chest syndrome complications.

**Conclusion:**

Sickle cell disease can be diagnosed by newborn screening but, as in this case, may have a delayed presentation. The delay in diagnosis of homozygous sickle cell disease illustrates that other genetic disorders should be considered in patients who already have a diagnosis of one Mendelian disorder but show atypical features.

## Introduction

Multiple molecular diagnoses may be more common than previously thought and occurred in ~ 4.9% of patients who were referred for whole genome sequence analysis [1].

Homozygous sickle cell disease (SS disease) is an autosomal recessive genetic disorder [2] with a variable phenotype and is common in many areas of the world [3]. Waardenburg syndrome type 4 (WS4) or Waardenburg-Shah syndrome is a rare disease globally, with less than 80 reported cases [4]. WS4 syndrome may be autosomal recessive or autosomal dominant, and a feature of this disorder is Hirschsprung’s disease [5].

We report a rare case of the co-inheritance of both SS disease and WS4 who presented to a specialist Sickle Cell Center (SCC). This case report is unusual as it is believed that this may be the first reported case to describe the blended phenotype of WS4 and SS disease and draws attention to the need for the further investigation of patients with one genetic disorder who present with atypical features. The report may raise awareness of the co-occurrence of SS disease and WS4.

## Case presentation

We present the case of an Afro-Caribbean woman who died at age 54 years. In addition to being mute her primary complaints on first presentation at age 7 were congenital deafness, jaundice, and constipation (Table 1). In addition, there was a history of snoring without mention of apnea. A history of similar congenital deafness and pigment abnormalities was reported in distant cousins. A half-sister who predeceased her had also been diagnosed as having SS disease. There was no previous history of pain or other sickle cell-related complications at this time.

**Table 1:** Timeline of major clinical events for case study

Dates	Relevant past medical history and interventions		
8-DEC-1965	This patient was known to have a history of Waardenburg syndrome. She had a family history of congenital deafness and pigment abnormalities. A half-sister who predeceased her had also been diagnosed with SS disease		
Dates	Summaries from initial and follow-up visits	Diagnostic testing (including dates)	Interventions
8-DEC-1965	Presented with jaundice. Diagnosed as having SS disease, in addition to Waardenburg syndrome	Hb electrophoresis	Referred to a specialist unit for sickle cell disease
8-JUN-1966	Acquired megacolon	Barium enema	High rectal biopsy
2-MAR-1967	Colon aganglionosis	–	Duhamel procedure
1978	Alopecia totalis	–	Hair pomade
7-NOV-1990	Leg ulcer	–	Regular dressing
9-MAR-2012	Bone pain crisis, suspected acute chest syndrome	Complete blood count	Buscopan (hyoscine butylbromide) 20 mg intramuscularly, Gravol (dimenhydrinate) 50 mg intramuscularly, and codeine 60 mg orally as “immediate” doses

*Hb* hemoglobin, *SS* disease homozygous sickle cell disease

**Fig. 1:**
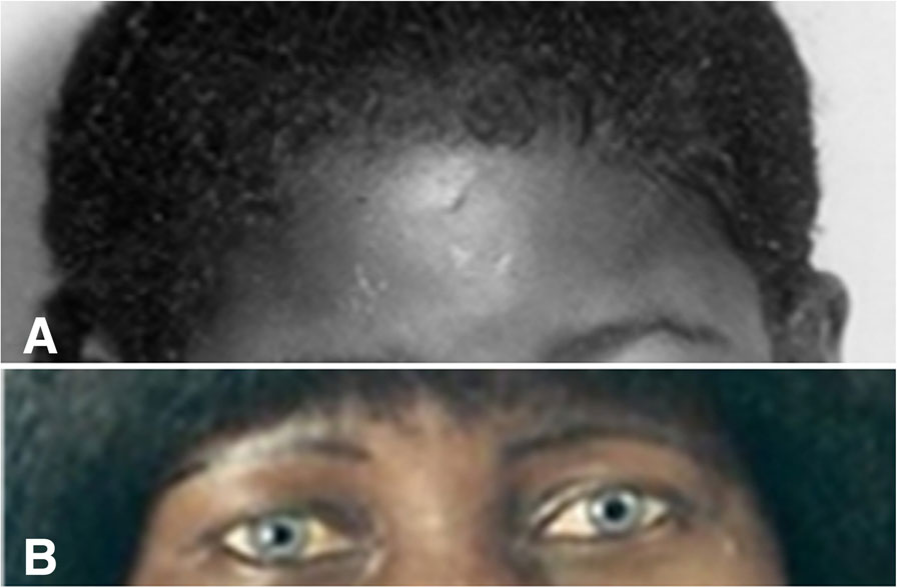
Patient as a child (**a**) and as an adult (**b**); note blue irides and icteric conjunctivae. Photo from childhood (**a**) was taken by the late Dr Paul Milner

On physical examination at first presentation at 7 years of age, she was afebrile and was found to be markedly icteric. She did not have a white forelock (Fig. 1) but was noted to be deaf, mute, and with blue eyes (Fig. 1) in keeping with her diagnosis of Waardenburg syndrome (WS). On abdominal examination, a hard mass was found; the dimensions were not described but it was thought to be a fecalith. In addition, her liver was documented as three fingers breadth below her right costal margin, a span was not documented. At that time she was diagnosed as having WS; acute cholestasis and SS disease were suspected. The diagnosis of SS was supported by a hemoglobin electrophoresis (Table 2), in addition to her previous diagnosis of WS. A later study revealed heterozygous deletional alpha thalassemia (Table 2), which is considered to be protective in persons with SS disease as persons with this trait tend to have less severe disease. At the time her hemoglobin level was 7.4. Of note, this varied from 7.5–8.2 g/dL up to an age of 39 years and fell to 6.0 g/dL in later years. Genetic testing for genetic mutations associated with WS4 was not possible because of availability. The results of hearing tests were not documented but she attended a school for the deaf and communicated by writing.

**Table 2:** Diagnostic assessments and recorded results for the patient in chronological order

Test	Age (years)	Results
Hemoglobin electrophoresis	7.8	SS disease
Complete blood count	8.0	Hb 7.5 g/dL, MCV 78 fl, retics 0.05 (proportion of red blood cells), WBC 24.0 × 10^9^/L, platelets 250 × 10^9^ /L
HbA_2_ range		3.7–4.3%
HbF range		1.2–4%
LE cells	8.7	Negative
Rectal biopsy	8.8	“Abundant muscle, numerous nerve fibers but no ganglion cells.”
Rectal biopsy (lowest value)	8.9	“No ganglion cells were seen. A few dubious nerve fibers were observed.”
Plain radiograph left hip	18.5	Unremarkable
Alpha globin gene number	26.9	Heterozygous deletional alpha thalassemia
Plain radiograph left hip	53.9	Sclerotic changes to head of femur and trochanteric area. Decreased joint space, osteophytic changes
Urea and electrolytes	53.9	K 5.8 mmol/L, creatinine 153 μmol/L (reference –males 80 μmol, females 68 μmol), Na^i^ 134 mmol/L, Cl^j^ mmol/L, HCO_3_^-^ 12 mmol/L
Complete blood count	54.0	Hb 6.0 g/dL, MCV 78 fl, retics 0.05 (proportion of red blood cells), WBC 12.0 × 10^9^/L, platelets 262 × 10^9^ /L. (Last SCC presentation 9 March 2012)

*Cl* chloride, *Hb* hemoglobin concentration, *HbA*_*2*_ hemoglobin A2, *HbF* fetal hemoglobin, *HCO*_*3*_^*-*^ bicarbonate, *K* potassium, *LE* lupus erythematosus, *MCV* mean cell volume, *Na* sodium, *retics* reticulocyte count, *SS disease* homozygous sickle cell disease, *SCC* Sickle Cell Center, *WBC* white cell count

Values given as a range are not dated

Surgical interventions included tonsilloadenoidectomy at 8 years due to upper airway obstruction. The post-surgery period was complicated with chest complications likely to have been acute chest syndrome. In addition, a Duhamel procedure bypassing the rectum was performed at age 9. This was preceded by prolonged history of recurrent presentations for constipation requiring fecal disimpaction under anesthesia. An acquired megacolon was diagnosed with a barium enema and total colonic aganglionosis had been diagnosed after two high rectal biopsies (Table 2) which led to the diagnosis of Hirschsprung’s disease with subsequent correction.

Follow-up visits at the SCC (Table 1) revealed other complaints which were mainly related to both WS and SS disease. Sickle cell-related complications included a bone pain episode which was first documented at 8 years of age. One to two bone pain crises were documented annually. She was treated mainly as an out-patient with acetaminophen, nonsteroidal anti-inflammatory agents, and occasionally opioids without adverse reaction. At her first presentation, pain and swelling of the dorsum of both hands were noted on physical examination and there was a limited range of movements at her wrists. A diagnosis of avascular necrosis was made. An important outcome of this was a shortened left fourth metacarpal diagnosed at 15 years. This is typical of infection complicating dactylitis (hand-foot syndrome). Although she complained of hip pain at 18 years of age, X-rays of her left hip showed no changes until 53.9 years when sclerotic changes suggestive of avascular necrosis were seen (Table 2).

At 18 years of age she complained of hair loss including eyebrows. As a result, she chose to wear a wig in adulthood (Fig. 1). She was referred to a dermatology clinic. Documented treatment included hair pomade. At 33 years she also complained of a chronic right leg ulcer and symptomatic gallstones in her later years requiring cholecystectomy.

At her last visit to the SCC, 3 weeks prior to her death, she complained of pain, cough, weight loss, and episodes of vomiting and was admitted to hospital with a diagnosis of acute chest syndrome and painful crisis. Her renal function was impaired (Table 2), and blood pressure was elevated (179/80 mmHg). Her hemoglobin was 4.5 gm/L and she was given a red blood cell transfusion and parenteral antibiotics. She later died in the intensive care unit.

## Discussion

The authors described a case of an Afro-Caribbean woman with co-inheritance of homozygous SS disease and WS. This case describes the features of this unusual phenotype.

Her morbidity pattern suggests that these diseases were inherited as separate disorders without overlap, and is referred to as a blended phenotype. The co-inheritance in this patient may be attributable to the worldwide distribution of both diseases. SS disease is common in the Caribbean, Africa, and Asia, with an incidence of 1 in 150 live births in Jamaica [6]. WS is seen in Northern Europe but the highest incidence is reported among Kenyan Africans [7].

This patient was atypical in the absence of a white forelock of hair or hair depigmentation and although alopecia is not a diagnostic criterion for WS4, congenital alopecia totalis has been reported with Hirschsprung’s disease [8]. Previously reported cases of WS4 have been diagnosed in the neonatal period [9, 10] and the Duhamel procedure in our case was performed relatively late.

Her age at death, 54 years, is above the median survival reported in a recent study of a Jamaican cohort [11], but lower than the 53 years for men and 59 years for women estimated from an earlier study [12]. The inheritance of alpha thalassemia trait and SS disease is associated with less severe disease [13, 14] and may have contributed to her relatively mild course in earlier years. Although sensorineural hearing loss may occur in SS disease [15], it is usually age related and not congenital as in WS4 syndrome. This was not considered to contribute to the congenital deafness experienced by our patient.

This report is limited by the inability to carry out whole exome genomic sequencing but, in the absence of this investigation, both diseases originate from very different molecular pathways and the phenotype in this case was typical of each disease.

## Conclusions

In conclusion, persons with a known molecular diagnosis need to be investigated if there are atypical findings. The delayed diagnosis of SS disease demonstrates that other genetic disorders should be considered in patients who already have the diagnosis of one Mendelian disorder but show atypical features.

## Abbreviations

SCC: Sickle Cell Center
SS disease: Homozygous sickle cell disease
WS: Waardenburg syndrome
WS4: Waardenburg syndrome type 4 or Waardenburg-Shah syndrome
